# *De Novo* Whole-Genome Sequencing of the Wood Rot Fungus *Polyporus brumalis*, Which Exhibits Potential Terpenoid Metabolism

**DOI:** 10.1128/genomeA.00586-17

**Published:** 2017-07-13

**Authors:** Su-Yeon Lee, Ji-eun An, Sun-Hwa Ryu, Myungkil Kim

**Affiliations:** Division of Wood Chemistry & Microbiology, Department of Forest Products, National Institute of Forest Science, Seoul, Republic of Korea

## Abstract

*Polyporus brumalis* is able to synthesize several sesquiterpenes during fungal growth. Using a single-molecule real-time sequencing platform, we present the 53-Mb draft genome of *P. brumalis*, which contains 6,231 protein-coding genes. Gene annotation and isolation support genetic information, which can increase the understanding of sesquiterpene metabolism in *P. brumalis*.

## GENOME ANNOUNCEMENT

Fungi are responsible for the production of many natural products via secondary metabolism through various biosynthetic pathways (1). *Polyporus brumalis* is a wood-degrading fungus that was shown to synthesize sesquiterpene compounds in liquid cultures with a simple carbohydrate source (2). Studies have shown that terpenes are biosynthesized through the mevalonate pathway, which is thought to originate from acetyl-coenzyme A (acetyl-CoA) produced during glycolysis (3). The biosynthesis of farnesyl pyrophosphate (FPP), which underlies the precursor of sesquiterpene, is produced from acetyl-CoA and requires eight enzymes. The sesquiterpene metabolites of *P. brumalis* have been confirmed to have a cyclic structure formed through cyclization reactions on its precursor molecule, FPP. The formation of the cyclic structure requires terpene synthase (TPS), which removes a phosphate group from the FPP and induces cyclization (4). However, there have been few studies on the genes and enzymes produced by wood rot fungi, limiting the understanding and engineering of biosynthetic processes. To obtain an overall view of terpene metabolism that occurs during fungal growth, it is necessary to identify as many genes as possible. Thus, this study was conducted to identify the genetic information associated with sesquiterpenoid metabolism of *P. brumalis*.

*De novo* genome sequencing of *P. brumalis* was conducted using PacBio single-molecule real-time sequencing technology. Genomic DNA was isolated from fresh mycelium obtained from Korean Collection for Type Cultures (KCTC46459). Draft genome sequencing obtained from the mycelium of *P. brumalis* confirmed that the genome size was approximately 56 Mb. A total of 676,520 reads were assembled using Canu software program (version 1.1), which resulted in 138 contigs, with an *N*_50_ value of 11,627. The cumulative G+C content of the genome assembly was 56.99%. Gene annotation was performed using the Maker program, and 6,231 protein-coding sequences were predicted in the draft genome. Unigenes were functionally characterized against the Gene Ontology (GO) and Kyoto Encyclopedia of Genes and Genomes (KEGG) databases. From the annotation results, the 15 unigenes encoding target 9 enzymes involved in sesquiterpene biosynthesis were discovered. To be specific, this study is focused on TPS, which may produce sesquiterpenes from provided starting substrates, such as geranyl pyrophosphate (GPP) and FPP. From the draft genome sequence data of *P. brumalis*, the putative TPS (unigene 00005412-RA) was found to have an amino acid sequence similar to the TPS found in *Dichotimus squalens*, *Tinea versicolor*, and *Gloeophyllum trabeum*. To understand the synthesis of specific sesquiterpene biosynthesis, we identified the full-length sequence of unigene 00005412-RA. The partial sequence of unigene 00005412-RA was extended toward the 5′ and 3′ ends using the SMART rapid amplification of cDNA ends (RACE) kit (Clontech, Mountain View, CA, USA), according to the manufacturer’s protocol. The gene contains a putative open reading frame of 1,185 bp that encodes 394 amino acids with a predicted molecular mass of 45.74 kDa and pI of 5.07.

### Accession number(s).

The *de novo* genome sequence of *P. brumalis* has been deposited in GenBank under accession number MARA00000000. The database of *P. brumalis* strain is available at http://112.220.192.2/pbr/. The full-length sequence was deposited with GenBank accession number AQH32583.
